# An Unusual Case of Marble Intraocular Foreign Body

**DOI:** 10.4103/0974-9233.53375

**Published:** 2008

**Authors:** Afekhide E. Omoti, Oseluese A. Dawodu, Osesogie U. Ogbeide

**Affiliations:** From the Department of Ophthalmology, University of Benin Teaching Hospital, Benin City, Nigeria

**Keywords:** marble, intraocular, foreign body, toxic

## Abstract

This report presents a case of marble intraocular foreign body that developed toxic complications during surgery. The patient is a 25 years old male who presented to the University of Benin Teaching Hospital with a history of trauma to the right eye while cutting marble. He was examined, had an ocular ultrasound scan and subsequently had an extracapsular cataract extraction. His visual acuity in the right eye was light perception. There was an entry point on the cornea, the lens was opaque, there was vitreous haemorrhage and the intraocular foreign body was localized in the posterior part of the posterior segment by ultrasound scan. He had extracapsular cataract extraction. During anterior capsulotomy, the cornea suddenly and rapidly became cloudy with a brownish tinge and the corneal epithelium started desquamating.Marble on its own may not be toxic but the other chemicals including cement, used in the processing of the marble were responsible for this delayed toxicity. Ultrasound scan is valuable in localisation of intraocular foreign bodies.

An intraocular foreign body (IOFB) is any material, organic or inorganic, which penetrates into the ocular tissue. This foreign material may be retained within the eye or it may be extruded out of the eye into the orbit, where it becomes intraorbital. Intraocular foreign bodies are important because they may result in poor vision and even loss of the eye. The complications of intraocular foreign bodies makes it a grave ophthalmic emergency.^1^ The foreign bodies may be classified as metallic or nonmetallic, with the metallic being divided into magnetic and nonmagnetic. They are also classified into toxic and nontoxic.^2^ Marble is nonmetallic and nontoxic.

A good history and ocular examination are still the most important in the diagnosis of intraocular foreign bodies.^3^ Radiological investigations such as plain X rays including the Combergs test or limbal ring sutured to the limbus, ocular ultrasonography, computed tomography and magnetic resonance imagery can be used in the localization of intraocular foreign bodies.^2^–^4^ Most intraocular foreign bodies are found in young adult males as a consequence of work accidents.^5^,^6^

Closed posterior intraocular microsurgery with vitreous instruments and bimanual surgical techniques have markedly altered the management of intraocular foreign bodies.^7^ Clinical features associated with better visual acuity outcomes include better presenting visual acuity, absence of clinical endophthalmitis, culture of a nonvirulent organism, lack of retinal detachment, shorter wound length, the size and type of the foreign body, minimal involvement of other intraocular structures and the timing of surgery.^8^,^9^

This report presents the case of a marble intraocular foreign body, which should otherwise be nontoxic but became toxic during surgery.

## Case Report

The patient is a 25years old male marble layer who presented to the eye clinic of the University of Benin Teaching Hospital on the 4th of April 2006 with a history of trauma and poor vision in the right eye of two weeks duration. He was using a machine to cut marble when a piece flew into his right eye resulting in severe pains, tearing and loss of vision. He used some traditional medication without relief.

He is not a known diabetic or hypertensive. There was no previous history of trauma to the eye or any other ocular disorder. He has no known drug allergy. He does not smoke but drinks alcohol occasionally.

**Figure 1:**
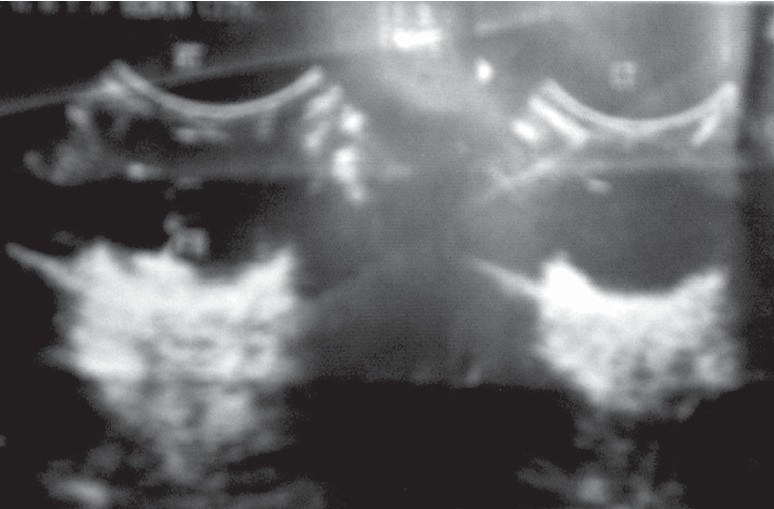
B scan of the eyeballs showing the intraocular foreign body in the posterior aspect of the vitreous cavity of the right eye.

His visual acuity was light perception in the right eye and 6/5 in the left eye. In the right eye, the conjunctiva was injected, there was a sealed corneal laceration of about 2.5mm representing the entry point of the foreign body at about the 2 o'clock position, the anterior chamber was shallow and there was some lens matter inferiorly. The pupil was mid-dilated, irregular, with posterior synechiae at about the 2 o'clock position. The lens was opaque. The fundus could not be visualized. The left eye was normal. The intraocular pressure, measured with the pulsair non-contact tonometer was 10mmHg in the right eye and 16mmHg in the left eye. An impression of sealed corneal laceration, traumatic cataract and retained intraocular foreign body in the right eye was made. Ocular ultrasound scan showed echogenic debris within the vitreous humour in the right eye suggestive of a vitreous hemorrhage and an acoustic shadow suggestive of a foreign body in the posterior aspect of the right eye (Fig 1). He was placed on topical steroids to suppress inflammatory reaction and topical cycloplegics to rest the eye.

He was subsequently booked for right extracapsular cataract extraction with a posterior chamber intraocular lens implant under local aneasthesia with the aid of an operating microscope. During the surgical procedure, the cornea rapidly became cloudy after anterior capsulotomy with loss of the surface epithelium of the cornea. The lens matter had a brownish colour and the cornea had a brownish tinge. Despite irrigation of the cortical lens matter with balanced salt solution, the posterior chamber intraocular lens could not be inserted because of poor visibility. The incision was sutured. He was given subconjunctival injections of dexamethasone, depomethyl prednisolone acetate and gentamicin, and chloramphenicol ointment applied before the eye was padded.

In the first postoperative day, the visual acuity in the right eye was light perception and the corneal haze had cleared a bit. Slit lamp examination showed a defect in the posterior capsule through which the vitreous haemorrhage could be visualised

## Discussion

The intraocular foreign body in this report occurred as an occupational hazard. The patient was cutting marble and was not wearing any eye protective device, neither was any provided by his employer. Such occupational hazards resulting in intraocular foreign bodies have been noted to occur most commonly in young adult males.^5^,^6^ In this study the patient affected is a 25 years old male. This is because most occupations in which people are exposed to injury are male dominated.^5^ It may also be related to greater activity in this group and hence greater exposure to the risk of injury.^6^

The entry point of the foreign body was through the cornea. It has been reported that the portal of entry of intraocular foreign bodies is predominantly corneal.^6^ The final location of the intraocular foreign body was in the posterior segment, either in the posterior vitreous or encroaching on the retina. Thus the piece of marble must have attained a high velocity to be able to penetrate the cornea, lens and end up in the posterior part of the eyeball. This high velocity may be responsible for the absence of clinically detectable secondary endophthalmitis since frictional heat may have sterilized the particle. However, this does not stop the marble particle from carrying other chemicals that may have been combined with it during processing. Thus, although the marble particle is classified as nontoxic, these other chemicals may be active and result in further chemical reactions and damage within the eyeball.

With the assumption that the marble intraocular foreign body was inert, the plan of management was to leave it alone and carry out an extracapsular cataract extraction and posterior chamber intraocular lens implant with the belief that this will restore vision to the eye, allow visualization of the intraocular foreign body, allow monitoring of the resolution of the vitreous haemorrhage and allow identification of any other tissue damage such as retinal tear or detachment. This line of management was supported by reports that early cataract extraction and intraocular lens implantation following penetrating eye wounds is justified and allows the patient to achieve a high visual acuity and is associated with a low rate of postoperative complications.^10^,^11^ Ordinarily, nontoxic substances such as non-oxidizable non-magnetic foreign bodies (such as marble) are preferably left in place.^12^ If the intraocular foreign body is toxic, a triple procedure comprising cataract extraction, posterior chamber intraocular lens implantation and vitrectomy combined in a one-stage procedure has been shown to be useful in the management of posterior segment intraocular foreign body associated with cataract.^13^–^15^ The technique of cataract extraction in this triple procedure may differ. It may vary from phacoemulsification with foldable intraocular lens implantation in the ciliary sulcus^13^ to pars plana lensectomy with anterior capsule preservation and intraocular lens implantation.^15^ The preserved anterior capsule provides support for the placement of an intraocular lens in the posterior chamber in the ciliary sulcus. In this center, facilities for phacoemulsification or lensectomy are not available. The patient was thus offered an extracapsular cataract extraction and posterior chamber intraocular lens implant. Closed posterior intraocular microsurgery with vitreous instruments and bimanual surgical techniques have markedly altered the management of intraocular foreign bodies.^7^,^13^–^15^ Unfortunately, facilities for these procedures are not available in our centre.

The eye was quiet before surgery but during capsulotomy, the cornea suddenly and rapidly became cloudy with a brownish tinge and there was desquamation of the surface corneal epithelium. This is because the marble was combined with other chemical substances during processing, most notably, cement. Capsulotomy may have caused a release of some of these substances, which may have been trapped within the crystalline lens or allowed some to sip in from the vitreous through the defect in the posterior capsule into the anterior chamber. Thus the marble, which should ordinarily be nontoxic, became toxic.

This case report further emphasizes the value of ocular ultrasound scan in the diagnosis of intraocular foreign bodies especially in developing countries in Africa, where other imaging techniques are either too expensive or not readily available.^16^
